# The Novel HDAC8 Inhibitor WK2-16 Attenuates Lipopolysaccharide-Activated Matrix Metalloproteinase-9 Expression in Human Monocytic Cells and Improves Hypercytokinemia In Vivo

**DOI:** 10.3390/ijms18071394

**Published:** 2017-06-29

**Authors:** Jing-Shiun Jan, Yung-Chen Chou, Yu-Wen Cheng, Chih-Kuang Chen, Wei-Jan Huang, George Hsiao

**Affiliations:** 1 Graduate Institute of Medical Sciences, College of Medicine, Taipei Medical University, Taipei 110, Taiwan; d119101004@tmu.edu.tw (J.-S.J.); d102094011@tmu.edu.tw (Y.-C.C.); 2 Department of Pharmacology, College of Medicine, Taipei Medical University, Taipei 110, Taiwan; 3 School of Pharmacy, College of Pharmacy, Taipei Medical University, Taipei 110, Taiwan; ywcheng@tmu.edu.tw; 4 Department of Physical Medicine and Rehabilitation, Chang Gung Memorial Hospital, Taoyuan 333, Taiwan; d118105008@tmu.edu.tw; 5 School of Medicine, Chang Gung University, Taoyuan 333, Taiwan; 6 Graduate Institute of Clinical Medicine, College of Medicine, Taipei Medical University, Taipei 110, Taiwan; 7 Graduate Institute of Pharmacognosy, Taipei Medical University, Taipei 110, Taiwan; 8 Ph.D. Program in Biotechnology Research and Development, College of Pharmacy, Taipei Medical University, Taipei 110, Taiwan

**Keywords:** histone deacetylase, matrix metalloproteinases-9 (MMP-9), lipopolysaccharide (LPS), endotoxemia

## Abstract

Dysregulated human monocytes/macrophages can synthesize and secrete matrix metalloproteinases (MMPs), which play important roles in the progression of sepsis. In this study, we investigated the effects and mechanism of a novel histone deacetylase (HDAC8) inhibitor, (*E*)-*N*-hydroxy-4-methoxy-2-(biphenyl-4-yl)cinnamide (WK2-16), on MMP-9 production and activation in stimulated human monocytic THP-1 cells. Our results demonstrated that the acetylation level of structural maintenance of chromosomes 3 (SMC3) was up-regulated by WK2-16 in THP-1 cells. Consistently, an in vitro enzyme study demonstrated that WK2-16 selectively inhibited HDAC8 activity. Moreover, the WK2-16 concentration dependently suppressed MMP-9-mediated gelatinolysis induced by tumor necrosis factor-α (TNF-α) or lipopolysaccharide (LPS). Additionally, WK2-16 significantly inhibited both MMP-9 protein and mRNA expression without cellular toxicity. Nevertheless, WK2-16 suppressed the extracellular levels of interleukin (IL)-6 from LPS-stimulated THP-1 cells. For the signaling studies, WK2-16 had no effect on LPS/TLR4 downstream signaling pathways, such as the NF-κB and ERK/JNK/P38 MAPK pathways. On the other hand, WK2-16 enhanced the recruitment of acetylated Yin Yang 1 (YY1) with HDAC1. Finally, in vivo studies indicated that WK2-16 could reduce the serum levels of TNF-α and IL-6 in endotoxemic mice. These results suggested that HDAC8 inhibition might provide a novel therapeutic strategy of hypercytokinemia in sepsis.

## 1. Introduction

Sepsis is defined as a life-threatening organ dysfunction caused by an abnormal host response to infection [1]. In the acute phase of severe sepsis, hypercytokinemia of the systemic cytokine storm may dysregulate the immune balance and enhance tissue damage and organ failure [2]. In intensive care units, septic patients have severely abnormal circulatory and cellular metabolism, which have been described as “sepsis shock” with particularly high mortality [1,3]. Although the administration of appropriate antibiotics and resuscitation therapies could improve the outcomes in septic injury [4,5,6], the survivors showed a poor prognosis [7]. Therefore, the development of the effective therapies for sepsis is an active field of clinical research.

Leukocytes, including monocyte/macrophages and neutrophils, play key roles in innate immunity to kill pathogens but cause tissue damage [8]. Because phagocytic cells are the major source of inflammatory mediators, such as tumor necrosis factor-α (TNF-α), interleukin (IL)-1β, IL-6 and matrix metalloproteinases (MMPs) [8,9,10], involved in the pathogenesis of sepsis. Additionally, the overproduction of MMPs could degrade the extracellular matrix that plays an important role in pathologic conditions, such as multiple sclerosis, arthritis and sepsis [11]. Recent studies have suggested that the serum levels of MMP-9 and tissue inhibitor of matrix metalloproteinase-1 (TIMP-1) are associated with mortality and organ injury [10,12,13,14]. Therefore, the reduction of the MMP-mediated inflammatory response may improve the outcome of sepsis.

Histone deacetylases (HDAC) and histone acetyl-transferases (HAT) both control chromatin remodeling and non-histone-related signaling [15]. HDAC inhibitors (HDACis) were clinically used for several diseases, such as cancer and rheumatoid arthritis [15,16,17]. Pan-HDACi, suberoylanilide hydroxamic acid (SAHA) was reported to attenuate the immune response and improve survival in a mouse model of cecal ligation and puncture [18], but this pan-HDACi showed some severe adverse effects in clinical uses [19]. HDAC8 is a unique class I HDAC that is primarily involved in the cohesion acetylation cycle. It could not influence global histone H3 acetylation, which is different from other class I HDACs in vivo [20,21,22]. HDAC8 was reported to regulate the acetylation of structural maintenance of chromosomes 3 (SMC3) and played a critical role in Cornelia de Lange syndrome [21,22,23]. In addition, HDAC8 affects multiple pathophysiological features, including influenza A virus endocytosis [24], the contraction of smooth muscle [25], and spindle assembly during mouse oocyte meiosis [26]. Furthermore, a recent report showed the HDAC8 inhibitor ITF3056 attenuated TNF-α and IL-1β production from lipopolysaccharide (LPS)-activated monocytes in vitro and the levels of cytokines in vivo [27]. However, the mechanism of HDAC8 inhibition on the production of inflammatory mediators has not been well described. 

LPS binds to the CD14/TLR4/MD2 receptor complex and activates downstream signaling molecules, including IκB kinase (IKK)/NF-κB and mitogen-activated protein kinase (MAPK), and then produces TNF-α and IL-1β, resulting in a severe inflammatory response [28]. Several transcription factors have also been described to be involved in MMP-9 expression, including NF-κB, AP-1, SP-1 and Ets [29]. MMP-9 could also be regulated negatively by transcription factor Yin Yang1 (YY1) in rat non-depolarized brain neurons [30,31]. However, it remains unclear whether HDAC8 inhibition influences the function of transcription factors on MMP-9 and pro-inflammatory cytokine expression in monocytic cells. 

In this study, we hypothesized that HDAC8 regulate inflammation-related MMP-9 and cytokine production from monocyte. Therefore, we investigated the effect and mechanism of a new structure and novel HDAC8 inhibitor, (*E*)-*N*-hydroxy-4-methoxy-2-(biphenyl-4-yl)cinna-mide (WK2-16), on MMP-9 production and activation in LPS-stimulated human monocytic THP-1 cells and its protective effects against hypercytokinemia in endotoxemic mice.

## 2. Results

### 2.1. WK2-16 Induces the Acetylation of SMC3 but Not the Global Histone H3 and α-Tubulin in THP-1 Cells

**Figure 1:**
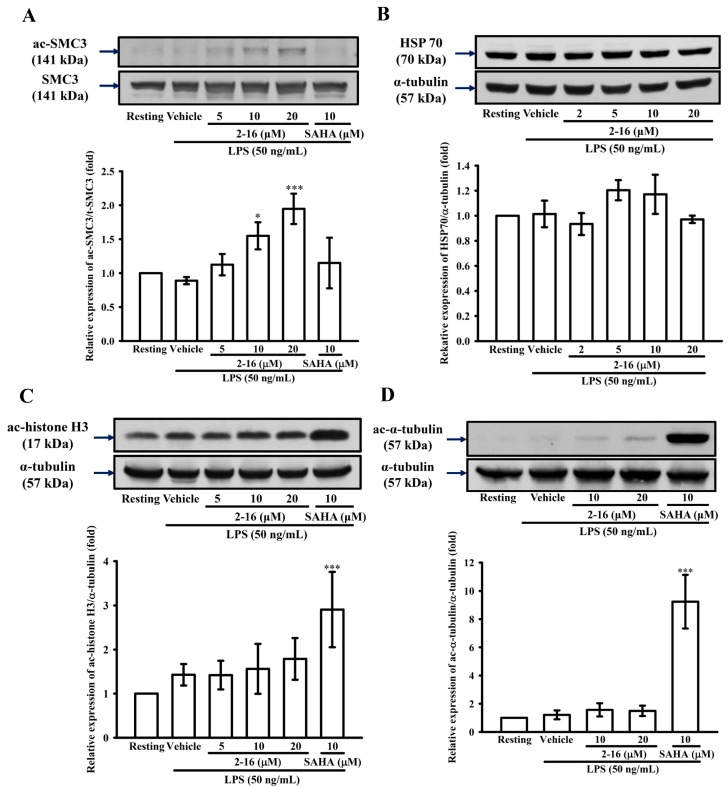
(*E*)-*N*-hydroxy-4-methoxy-2-(biphenyl-4-yl)cinnamide (WK2-16) induces the hyperacetylation of structural maintenance of chromosomes 3 (SMC3), but did not have a significant effect on acetyl-histone-H3, acetyl-α-tubulin and heat shock protein 70 (HSP70) expression. Human monocytic THP-1 cells (10^6^ cells/mL) were dispensed onto six-well plates and were treated with WK2-16 (5, 10 and 20 μM), suberoylanilide hydroxamic acid (SAHA) (10 μM) or vehicle for 15 min followed by treatment with lipopolysaccharide (LPS) (50 ng/mL) for: 2 h (**A**,**B**); or 30 min (**C**,**D**). Cell lysates were obtained and analyzed for the acetylation of: SMC3 (**A**); HSP70 (**B**); α-tubulin (**C**); and histone-H3 (**D**) by Western blotting. The data are represented as the means ± S.D. from three to five independent experiments. * *p* < 0.05, *** *p* < 0.001 compared with the vehicle.

To determine whether WK2-16 selectively inhibited HDAC8 activity in LPS-stimulated THP-1 cells, the level of acetylation of the intracellular targets of HDACs, such as SMC3, histone H3 and α-tubulin, was evaluated by immunoblotting (Figure 1). THP-1 cells were pretreated with WK2-16 (5, 10 and 20 μM), the pan-HDAC inhibitor SAHA (10 μM) or vehicle (DMSO) for 15 min followed by the addition of LPS (50 ng/mL). It was found that WK2-16 significantly induced the levels of acetylated SMC3, the specific substrate of HDAC8 [32], by 1.12 ± 0.16, 1.55 ± 0.20, and 1.95 ± 0.22-fold compared with those under the normal condition, respectively (Figure 1A). It was reported that class I HDACI inhibition enhances anti-inflammatory chaperone heat shock protein 70 (HSP70) expression [33,34,35]. However, WK2-16 did not up-regulate HSP70 expression (Figure 1B). Furthermore, SAHA strongly enhanced the levels of acetylated histone H3 and α-tubulin, respectively. WK2-16 had no significant effect on the levels of acetylated histone H3 and α-tubulin compared with that in the normal control and LPS-stimulated groups (Figure 1C,D). The IC_50_ of WK2-16 with HDAC8 inhibition was found to be 126.3 ± 6.4 nM in an in vitro enzyme study, a level that was more potent than SAHA at 4160.3 ± 14.2 nM (*n* = 3, data not shown). According to these results, WK2-16 inhibited HDAC8 activity with a higher selectivity.

### 2.2. The Effects of WK2-16 on MMP-9-Mediated Gelatinolysis Induced by LPS, TNF-α or PMA and MMP-2-Mediated Gelectinolysis by TGF-β

**Figure 2:**
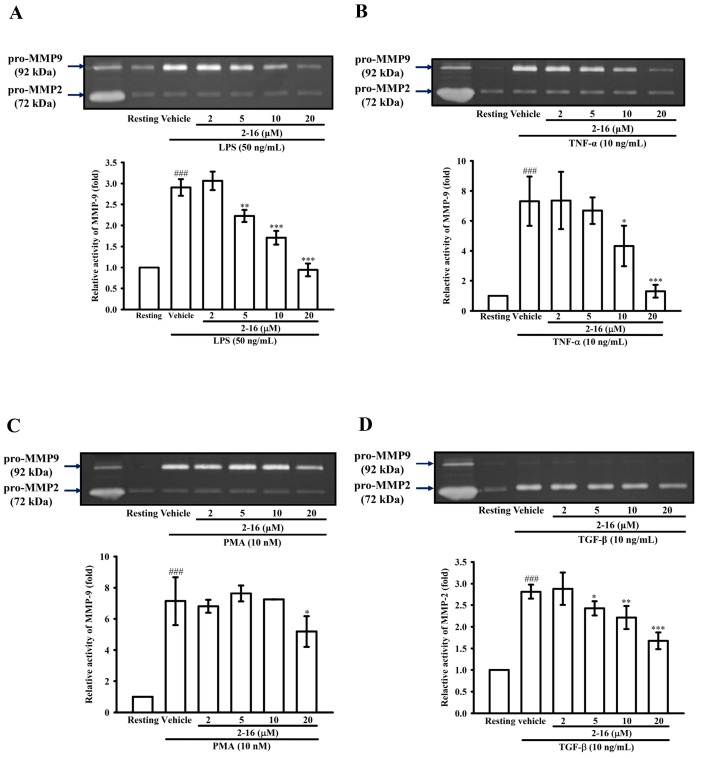
WK2-16 suppresses matrix metalloproteinase (MMP)-9- and MMP-2-mediated gelatinolysis induced by different stimulants. THP-1 cells (5 × 10^5^ cells/0.5 mL) were dispensed onto 24-well plates and were treated with: LPS (50 ng/mL) (**A**); tumor necrosis factor (TNF)-α (10 ng/mL) (**B**); phorbol 12-myristate 13-acetate (PMA) (10 nM) (**C**); or transforming growth factor (TGF)-β (10 ng/mL) (**D**) for 24 h as indicated. THP-1 cells were treated with the indicated concentrations of WK2-16 (2, 5, 10 and 20 μM) or vehicle or 15 min before treatment with stimulant. The cell-free supernatants were then assayed for MMP activity by gelatin zymography. The data are represented as the means ± S.D. from three to four independent experiments. ### *p* < 0.001 compared with the resting condition. * *p* < 0.05, ** *p* < 0.01 and *** *p* < 0.001 compared with the vehicle.

To investigate whether WK2-16 has any effect on the production of MMP-9 or MMP-2 induced by stimulants, the culture medium of different stimulants activated THP-1 cells was assessed by zymography. THP-1 cells were pretreated with WK2-16 (2, 5, 10 and 20 μM) for 15 min followed by the addition of LPS (50 ng/mL), TNF-α (10 ng/mL), phorbol 12-myristate 13-acetate (PMA) (10 μM) or Transforming growth factor (TGF)-β (10 ng/mL). As shown in Figure 2A, compared with the resting condition, LPS (50 ng/mL) significantly enhanced extracellular MMP-9-mediated gelatinolysis by up to 3.06 ± 0.12-fold, and pretreatment with WK2-16 (5, 10 and 20 μM) strongly suppressed MMP-9-mediated gelatinolysis in a concentration-dependent manner by 2.26 ± 0.14, 1.71 ± 0.40, and 0.99 ± 0.36-fold, respectively. Similarly, TNF-α (10 ng/mL) significantly increased extracellular MMP-9 gelatinolysis by up to 7.32 ± 1.65-fold compared with that under the resting condition. Pretreatment with WK2-16 (5, 10 and 20 μM) significantly reduced extracellular MMP-9 gelatinolysis in a concentration-dependent manner by 6.68 ± 0.89, 4.33 ± 1.35, and 1.31 ± 0.03-fold, respectively (Figure 2B). Pretreatment with WK2-16 (20 μM) partially suppressed PMA (10 nM)-induced extracellular MMP-9 gelatinolysis (Figure 2C). Furthermore, pretreatment with WK2-16 (5, 10 and 20 μM) partially suppressed MMP-2-mediated gelatinolysis induced by TGF-β (10 ng/mL) in a concentration-dependent manner by 2.43 ± 0.16, 2.21 ± 0.27, and 1.68 ± 0.19-fold, respectively (Figure 2D).

### 2.3. WK2-16 Inhibits the LPS-Induced Expression of Intracellular MMP-9 Protein and mRNA 

**Figure 3:**
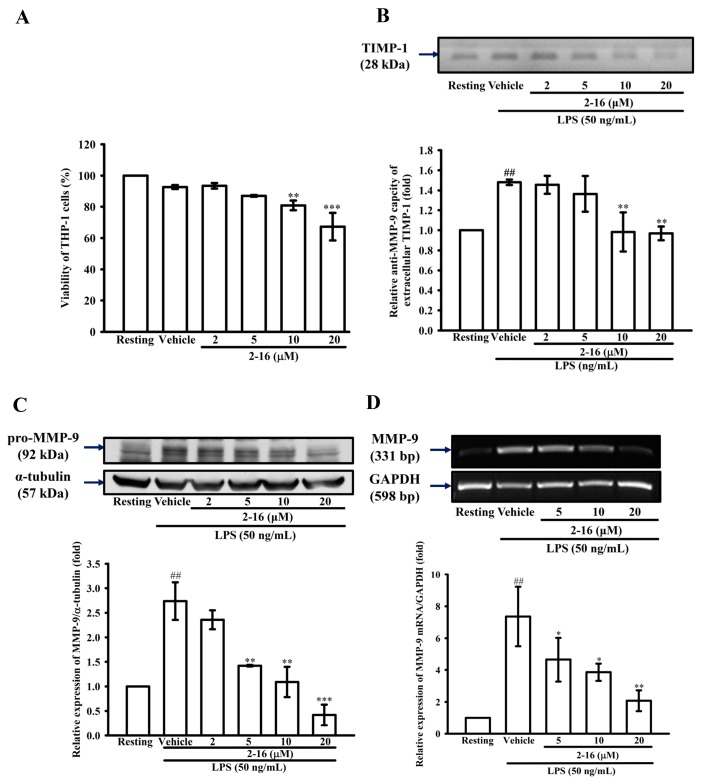
WK2-16 inhibits MMP-9 protein and mRNA expression induced by LPS in THP-1 cells without cellular toxicity. (**A**) THP-1 cells (5 × 10^5^ cells/0.5 mL) were dispensed onto 24-well plates and were treated with the indicated concentrations of WK2-16 (2, 5, 10 and 20 μM) or vehicle for 24 h. Cell viability was quantified by the ability of mitochondria to reduce the tetrazolium dye 3-(4,5-dimethylthiazol-2-yl)-2,5-diphenyl tetrazolium bromide (MTT) in viable cells. (**B**) The activity of tissue inhibitor of matrix metalloproteinase-1 (TIMP-1) as assessed by reverse zymography of conditioned media from THP-1 cells treated with the indicated concentrations of WK2-16 (2, 5, 10 and 20 μM) or vehicle or 15 min before exposure to LPS (50 ng/mL) for 24 h. (**C**,**D**) THP-1 cells (10^6^ cells/mL) were dispensed onto six-well plates and were treated with LPS (50 ng/mL) for: 24 h (**C**); or 8 h (**D**) at the indicated concentrations of WK2-16 (2, 5, 10 and 20 μM) or vehicle for 15 min before treatment with LPS. Cell lysates were obtained and analyzed for MMP-9 protein expression by Western blotting or for MMP-9 mRNA expression by RT-PCR. The data are represented as the means ± S.D. from three independent experiments. ## *p* < 0.01 compared with the resting condition, * *p* < 0.05, ** *p* < 0.01 and *** *p* < 0.001 compared with the vehicle.

According to the MTT assay, WK2-16 had only a partial effect on cell viability at the high concentrations (10 μM with 80.82 ± 3.09% and 20 μM with 67.26 ± 8.78%) (Figure 3A). To confirm whether WK2-16 down-regulates extracellular MMP-9 gelatinolysis by the up-regulation of extracellular TIMP1, THP-1 cells were pretreated with WK2-16 (5, 10 and 20 μM) for 15 min followed by the addition of LPS (50 ng/mL) for 24 h. Reverse zymography showed that THP-1 cells constitutively releasing TIMP-1 were enhanced by LPS stimulation that were suppressed by WK2-16 pretreatment (Figure 3B). Furthermore, to determine whether WK2-16 inhibited extracellular MMP-9 gelatinolysis through the regulation of MMP-9 expression, MMP-9 expression was evaluated by immunoblotting, and MMP-9 mRNA was evaluated by RT-PCR. As shown in Figure 3C, compared with the resting condition, THP-1 cells stimulated with LPS for 24 h significantly increased MMP-9 protein expression by up to 2.74 ± 0.38-fold. Pretreatment with WK2-16 (2, 5, 10 and 20 μM) for 15 min before LPS demonstrated that WK2-16 concentration dependently suppressed MMP-9 expression down to 2.36 ± 0.19, 1.42 ± 0.02, 1.09 ± 0.31, and 0.42 ± 0.21-fold compared with that under the normal condition, respectively. Similarly, LPS (50 ng/mL) significantly increased the expression of MMP-9 mRNA in THP-1 cells by up to 6.83 ± 1.11-fold compared with that under the normal condition, and pretreatment with WK2-16 (5, 10 and 20 μM) significantly attenuated LPS-induced MMP-9 mRNA expression (Figure 3D). These results suggested that WK2-16 down-regulated MMP-9-mediated gelatinolysis occurred at the transcriptional level.

### 2.4. WK2-16 Down-Regulates IL-6 Levels but Enhances Cyclooxygenase (COX)-2 Expression

**Figure 4:**
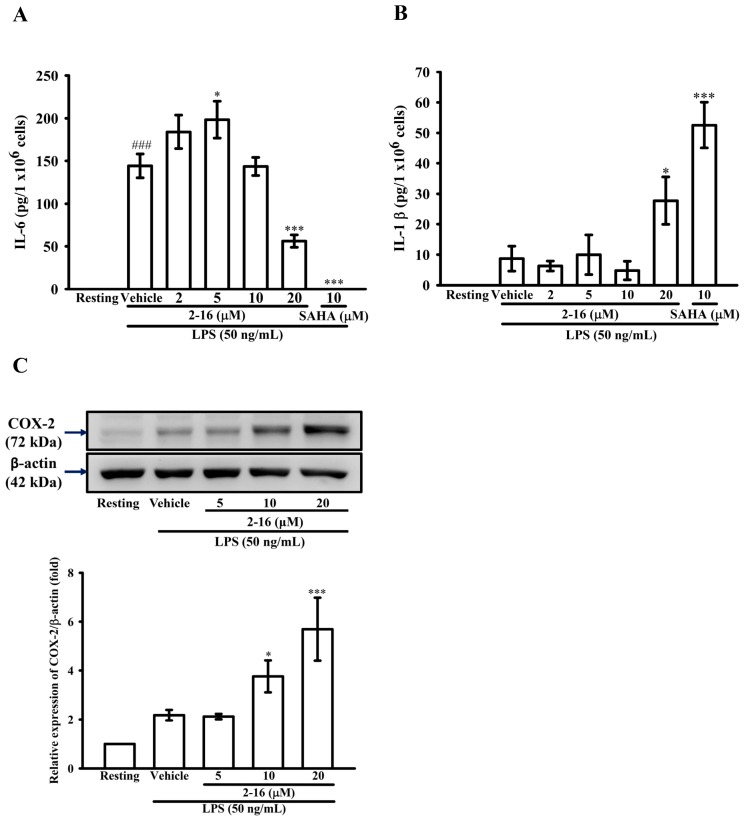
Effect of WK2-16 on LPS-induced cytokine production and cyclooxygenase (COX)-2 expression in THP-1 cells. (**A**,**B**) THP-1 cells (5 × 10^5^ cells/0.5 mL) were dispensed onto 24-well plates and were treated with the indicated concentrations of WK2-16 (2, 5, 10 and 20 μM), SAHA (10 μM) or vehicle for 15 min followed by treatment with LPS (50 ng/mL) for 24 h. Cell-free supernatants were then assayed for the levels of: interleukin (IL)-6 (**A**); and IL-1β (**B**) by ELISA. The data were represented as the means ± S.D. from three independent experiments. ### *p* < 0.01 compared with the resting condition, * *p* < 0.05 and *** *p* < 0.001 compared with the vehicle. (**C**) THP-1 cells (10^6^ cells/mL) treated with LPS (50 ng/mL) for 24 h at the indicated concentrations of WK2-16 (2, 5, 10 and 20 μM) or vehicle for 15 min before treatment with LPS. Cell lysates were obtained and analyzed for COX-2 by WB. The data are represented as the means ± S.D. from three independent experiments. * *p* < 0.05 and *** *p* < 0.001 compared with the vehicle.

To investigate whether the WK2-16 regulates the production of the pro-inflammatory cytokines IL-1β, IL-6, and COX-2 in activated THP-1 cells, the latter was pretreated with WK2-16 (5, 10 and 20 μM) for 15 min followed by the addition of LPS (50 ng/mL) within 24 h. As shown in Figure 4A, compared with the resting condition, LPS significantly induced extracellular IL-6 levels by up to 144.2 ± 13.9 pg/10^6^ cells, and pretreatment with WK2-16 (10 and 20 μM) significantly suppressed the extracellular IL-6 levels in a concentration-dependent manner to 143.5 ± 10.6, and 56.1 ± 7.3 pg/10^6^ cells, respectively (Figure 4A). However, similar to the pretreatment of SAHA (10 μM) significantly enhancing extracellular IL-1β levels to 52.6 ± 7.5 pg/10^6^ cells, the pretreatment of WK2-16 (20 μM) also enhanced extracellular IL-1β levels to 27.7 ± 7.8 pg/10^6^ cells (Figure 4B). On the other hand, as shown in Figure 4C, pretreatment with WK2-16 (10 and 20 μM) concentration dependently increased COX-2 protein expression by up to 2.7 ± 0.4 and 5.7 ± 1.3-fold, respectively (Figure 4C).

### 2.5. The Effects of WK2-16 on NF-κB Signaling Induced by LPS in THP-1 Cells

**Figure 5:**
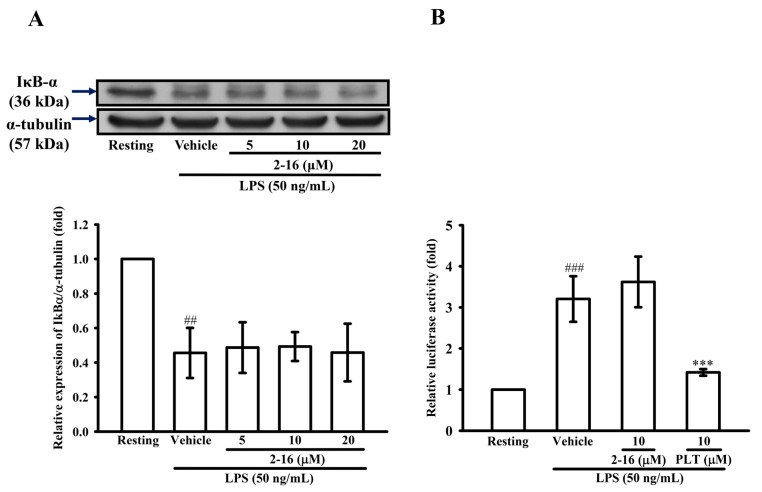
WK2-16 does not influence NF-κB activation induced by LPS in THP-1 cells. (**A**) THP-1 cells (1 × 10^6^ cells/mL) were dispensed onto six-well plates and were treated with WK2-16 (2, 5, 10 and 20 μM) or vehicle for 15 min followed by treatment with LPS (50 ng/mL) for 75 min. Cell lysates were obtained and analyzed for IκB-α protein stability by WB. (**B**) THP-1 cells were treated with WK2-16 (10 μM), parthenolide (PTL, 10 μM) or vehicle for 15 min followed by treatment with LPS (50 ng/mL) for 4 h. Cell lysates were obtained and analyzed for NF-κB activation by the luciferase assay. The data were represented as the means ± S.D. from three independent experiments. ## *p* < 0.01 and ### *p* < 0.001 compared with the resting condition, *** *p* < 0.001 compared with the vehicle.

NF-κB, AP-1, SP-1 and Ets control MMP-9 expression, as has been described previously [29]. Thus, the effect of WK2-16 on the LPS-induced degradation of the inhibitor of κ B α (IκBα) was evaluated by WB. As shown in Figure 5A, significant degradation of IκBα was observed in THP-1 exposed to LPS (50 ng/mL) for 30 min. However, the LPS-induced degradation of IκBα was not prevented by the pretreatment of WK2-16 (20 μM). Thus, to confirm whether WK2-16 directly regulated NF-κB-binding activity, the luciferase assay was used. As shown in Figure 5B, compared with the resting condition, NF-κB-dependent luciferase gene expression was strongly induced by LPS to 3.21 ± 0.55-fold at 4.5 h, and pretreatment with an IKK inhibitor, parthenolide (PTL, 10 μM), strongly reduced LPS-induced reporter gene activity to 1.42 ± 0.08-fold in LPS-stimulated THP-1 cells. However, unlike parthenolide, pretreatment with WK2-16 (10 μM) did not influence NF-κB-dependent reporter gene activity. 

### 2.6. The Effects of WK2-16 on the LPS-Induced MAPK Activation in THP-1 Cells

**Figure 6:**
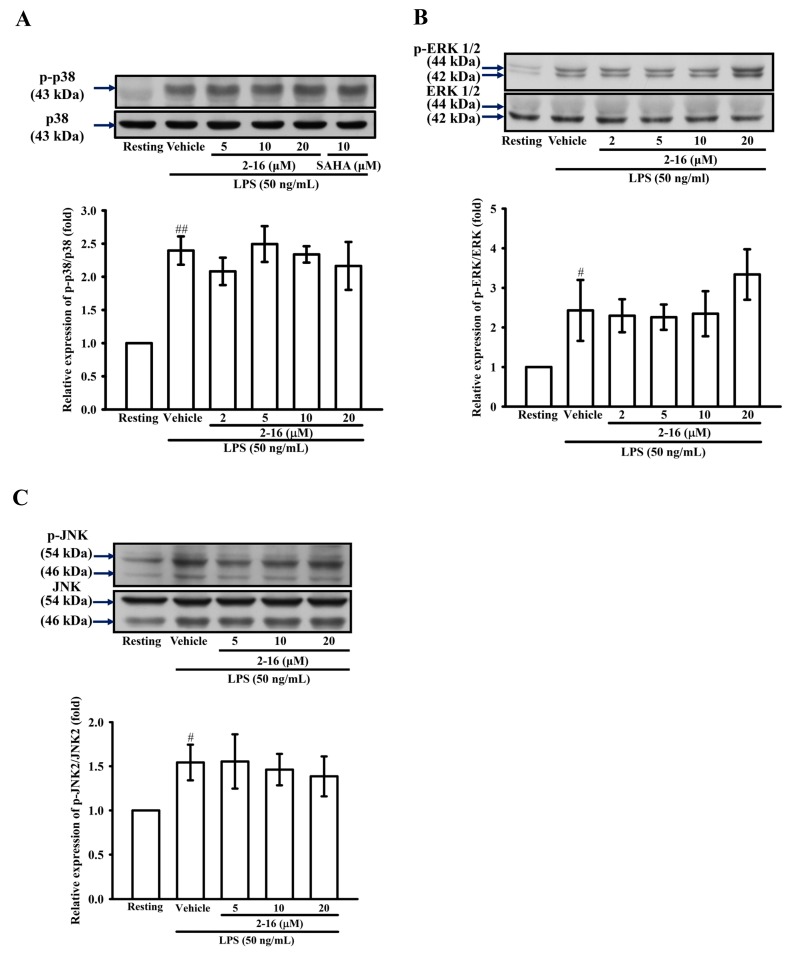
WK2-16 has no effect on the activation of the MAPK pathway induced by LPS in THP-1 cells. (**A**) THP-1 cells (1 × 10^6^ cells/mL) were dispensed onto six-well plates and were treated with WK2-16 (2, 5, 10 and 20 μM) or vehicle for 15 min followed by treatment with LPS (50 ng/mL) for 90 min. Cell lysates were obtained and analyzed for p38 phosphorylation by WB. (**B**) THP-1 cells were treated with WK2-16 (2, 5, 10 and 20 μM) or vehicle for 15 min followed by treatment with LPS (50 ng/mL) for 30 min. Cell lysates were obtained and analyzed for ERK phosphorylation by Western blotting. (**C**) THP-1 cells were treated with WK2-16 (5, 10, 20 μM) or vehicle for 15 min followed by treatment with LPS (50 ng/mL) for 45 min. Cell lysates were obtained and analyzed for JNK phosphorylation by WB. The data are represented as the means ± S.D. from three independent experiments. # *p* < 0.05 and ## *p* < 0.01 compared with the resting condition.

To determine the effect of WK2-16 on the LPS-induced activation of MAPKs in THP-1 cells, the phosphorylation levels of p38, ERK and JNK MAPKs were evaluated by WB. As shown in Figure 5, compared with the resting condition, THP-1 cells exposed to LPS (50 ng/mL) for different durations were identified in previous studies [36], and LPS significantly enhanced the phosphorylation of p38, ERK and JNK MAPKs by 2.40 ± 0.41, 2.43 ± 0.17, and 1.54 ± 0.20-fold. However, pretreatment with various concentrations of WK2-16 did not affect the phosphorylation of p38, ERK and JNK MAPKs (Figure 6A–C, respectively). 

### 2.7. WK2-16 Enhances the Recruitment of YY1/HDAC1 in LPS-Stimulated THP-1

**Figure 7:**
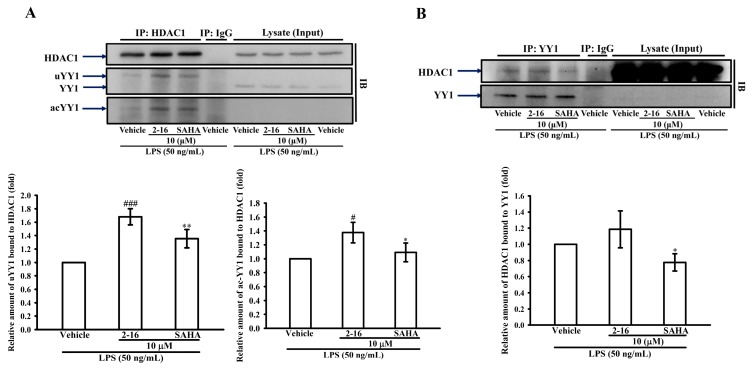
WK2-16 induces the recruitment of Yin Yang 1 (YY1) and HDAC1 in LPS-stimulated THP-1 cells. THP-1 cells (1.2 × 10^7^ cells/12 mL) were dispensed on 10-cm dishes and were treated with WK2-16 (10 μM), SAHA (10 μM) or vehicle for 15 min followed by treatment with LPS (50 ng/mL) for 2 h. Cell lysates were obtained, and 1 mg of proteins from each dish was subjected to Co-IP with: anti-HDAC1 antibody (IP: HDAC1) (**A**); or anti-YY1 antibody (IP: YY1) (**B**) and was analyzed for HDAC1, YY1, ubiquitinated YY1 (uYY1, the super shift of IP: YY1) and acetylated YY1 (acYY1) expression by WB (IB). As a negative control (Ig G), 1 mg of proteins from cell lysates was immunoprecipitated with anti-IgG antibody. In the input lane, 30 μg of proteins of cell lysates was loaded. The data are represented as the means ± S.D. from three independent experiments. # *p* < 0.05 and ### *p* < 0.001 compared with the vehicle, * *p* < 0.05 and ** *p* < 0.01 compared with WK2-16 treatment.

The transcriptional regulator Yin Yang 1 (YY1) plays an important role in MMP-9 expression in brain neurons [30,31]. Furthermore, it was reported that HDAC8 inhibition represses the transcriptional activity of YY1 [37]. To determine whether WK2-16 inhibits MMP-9 expression through regulating YY1, the protein association of YY1 was evaluated by Co-IP. THP-1 cells were pretreated with WK2-16 (10 μM), SAHA (10 μM) or vehicle (DMSO) for 15 min followed by the addition of LPS (50 ng/mL) for 2 h. Interestingly, the pretreatment of WK2-16 significantly enhanced the relative amount of ubiquitinated-YY1 (uYY1) and acetylated-YY1 (acYY-1) bound to HDAC1 by 1.68 ± 0.12 and 1.38 ± 0.15-fold compared with that in the vehicle control, respectively (Figure 7A). In addition, unlike SAHA pretreatment significantly reducing the amount of YY1 bound to HDAC1 (28.7%), WK2-16 did not down-regulate the association of HDAC1/YY1 (Figure 7B). These results suggested WK2-16 inhibited MMP-9 expression by enhancing the recruitment of ubiquitination-YY1/acetylated-YY1 and HDAC1 on the MMP-9 promoter.

### 2.8. WK2-16 Attenuates Both the Levels of IL-6 and TNF-α in Septic Mice

**Figure 8:**
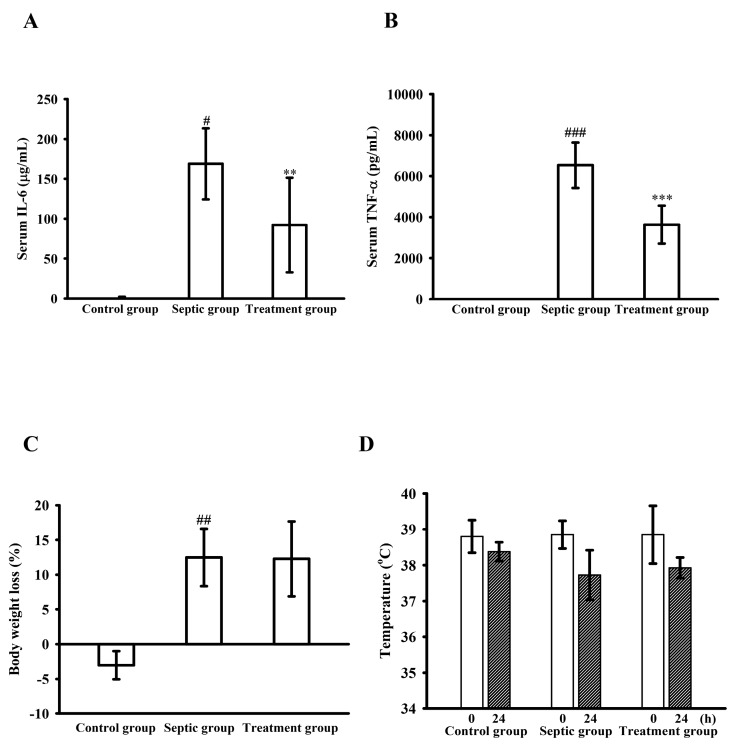
WK2-16 reduces cytokine production during endotoxemia in vivo. C57BL/6 mice were treated intraperitoneally with WK2-16 (30 mg/kg, treatment group, *n* = 4) or an equivalent volume of the vehicle solution for 30 min before the injection of LPS (10 mg/kg, septic group, *n* = 4) or a placebo (control group, *n* = 4). The whole blood was collected via submandibular bleeding at the indicated times. The serum levels of: IL-6 (**A**) (2 h); and tumor necrosis factor (TNF)-α (**B**) (1 h) were quantified by ELISA. The body weight difference (**C**); and body temperature loss (**D**) were compared with those of the mice before and after 24-h treatment, respectively. The data were represented as the means ± S.D. # *p* < 0.05, ## *p* < 0.01 and ### *p* < 0.001 compared with the control group, ** *p* < 0.01 and *** *p* < 0.001 compared with the septic group.

LPS-induced endotoxemia was used to examine the in vivo effect of WK2-16 on pro-inflammatory cytokine production. Male C57BL/6 mice were administered intraperitoneally with WK2-16 (30 mg/kg) or vehicle for 30 min before LPS (10 mg/kg) injection. As shown in Figure 8A, LPS injection for 2 h significantly induced plasma IL-6 levels to 168.9 ± 44.6 μg/mL, and pretreatment with WK2-16 strongly attenuated IL-6 production to 92.0 ± 59.3 μg/mL compared with the septic group (Figure 8A). Furthermore, WK2-16 significantly attenuated the serum IL-6 level to 14.9 ± 17.8 pg/mL compared with that in the septic group (56.0 ± 38.4 pg/mL) at 24 h (data not shown). Similarly, serum TNF-α levels were significantly increased to 6532.4 ± 1110.5 pg/mL, and pretreatment with WK2-16 significantly reduced the plasma TNF-α level to 3632.1 ± 927.2 pg/mL at 1 h (Figure 8B). However, WK2-16 could not prevent body weight loss (Figure 8C) and body temperature loss compared with 24 h before (Figure 8D) of endotoxemic mice. These results suggested that WK2-16 also suppressed IL-6 and TNF-α secretion induced by LPS in vivo*.*

## 3. Discussion

Although the Sequential Organ Failure Assessment score could be used to fast screen patients with intermediate risk of sepsis progression, there is only the supportive treatment which could not improve organ failure [1]. In the other hands, MMP-9 also has been reported to be a useful diagnostic biomarker of the severity of sepsis [38,39,40]. In addition, MMP-9 contributes to the progression of septic-associated encephalopathy [41], myocardial depression [42] and is associated with a rising blood glucose level in patients with early sepsis [43]. This study suggested the possible mechanism underlying how WK2-16 suppresses LPS-mediated MMP-9 expression via regulating the acetylation levels of YY1/HDAC1 complex; however, it is NF-κB independent. Furthermore, WK2-16 attenuates the TNF-α and IL-6 protein levels in endotoxemic mice.

HDAC8 has not been shown to deacetylate core histone that is different from other member of class I HDACs. Previous studies have shown HDAC8 regulates multiple non-histone substrates, such as SMC3, ERRα and p53 [22]. HDAC8 inhibition leads to the enhanced acetylation of SMC3 and p53 [44] but not to the induction of global histone H3 and α-tubulin hyperacetylation [20]. Similarly, our results showed that WK2-16 induces SMC3 hyperacetylation but does not significantly increase global histone H3 and the α-tubulin acetylation level in LPS-stimulated THP-1 cells at less than 10 μM, whereas the pan-HDAC inhibitor SAHA robustly acetylates α-tubulin. Although it has been reported that class I HDAC inhibition induces HSP 70 expression, which attenuates neuroinflammation induced by LPS in vitro and in vivo [33,34,35], WK2-16 did not influence HSP70 expression in LPS-stimulated THP-1 cells even 20 μM. Taken together, these results support that WK2-16 selectively inhibits HDAC8 activity [45].

Intracellular class I HDAC inhibition represses MMP-2 and MMP-9 expression, an activity that has been described previously [46]. However, it remains unclear whether HDAC8 regulates MMP-9 production. The activity of *Mmp9* is regulated by NF-κB, AP-1, SP-1 and Ets, as that had been described previously [29]. LPS [36], TNF-α [33], PMA [47] and TGF-β [48] have been used in this study to investigate whether WK2-16 influences multiple MMP production pathways. Our results demonstrated that WK2-16 significant suppresses both LPS- and TNF-α-induced MMP-9 gelatinolysis in a concentration-dependent manner, and WK2-16 (20 μM) partially suppresses either PMA-induced MMP-9 or TGF-β-induced MMP-2 gelatinolysis in THP-1 cells. Furthermore, WK2-16 concentration dependently repressed LPS-stimulated MMP-9 protein and mRNA expression without cellular toxicity, but did not enhance TIMP-1 activity.

HDAC8 inhibition has different effects on pro-inflammatory cytokine production. Li et al. reported that the HDAC8 inhibitor ITF3056 attenuated TNF-α and IL-1β production without a significant decrease in the IL-1β mRNA levels in vitro [27]. However, another study described that HDAC8 could suppress IL-1β production, leading to the hypoacetylation of histone H3K27 located on the pro-IL-1β enhancer and promoter regions in macrophages, such function that could be reversed by the HDAC8 inhibitor PCI-34051 to increase IL-1β production [49]. Consistently, the function of WK2-16 on IL-1β production is similar to PCI-34051. Our results showed that WK2-16 could suppress the extracellular IL-6 levels induced by LPS, but enhanced the IL-1β extracellular production of LPS-stimulated THP-1 cells at the high concentration (20 μM). Therefore, the detailed mechanism underlying HDAC8 inhibition regulates IL-1β production and secretion needs to be further investigated.

Previous studies have shown that pan-HDACi SAHA suppresses NF-κB p65 nuclear accumulation [50], and manipulation of HDAC1/2/3 silencing reduces p38 phosphorylation to attenuate pro-inflammatory cytokine secretion [51]. Although these pathways have been proven to be both involved in MMP-9 and COX-2 gene expression, WK2-16 as HDAC8 inhibitor had no effect on IκB-degradation, the transactivation of NF-κB and the activation of ERK/JNK/p38 MAPK pathways induced by LPS in THP-1 cells. On the other hand, HDAC8 inhibition was reported to down-regulate the transcriptional activity of YY1 [37]. Furthermore, YY-1 could suppress MMP-9 expression [30,31] or enhance COX-2 expression [52]. According to the different effect of WK2-16 on MMP-9 and COX-2 expression, we proposed that it might influence the function of YY-1.

Multifunctional transcription factor YY1 is involved in gene expression and repression. YY1 may lead to activator displacement, regulate the activator functions or recruit corepressors to suppress target gene expression [53,54]. The transcriptional activity of YY1 is regulated by acetylation and self-ubiquitination, and the hyperacetylation of the central residues 170–200 of YY1 enhances YY1-mediated gene suppression [53,54]. YY1 binds Class I HDACs and regulates its own activity, which is important in YY1-mediated gene suppression [54,55]. In addition, YY1 is bound to the MMP-9 gene and recruits HDAC3 to deacetylate histones H3 and H4, leading to the condensation of chromatin around the MMP-9 promoter [30,31]. Our results demonstrated that WK2-16 increased acetylated and ubiquitinated YY1 bound to the HDAC1 complex. By contrast, the pan-HDAC inhibitor SAHA significantly decreases the interaction of YY1 and HDAC1. However, there is no interaction between HDAC8/YY1 and HDAC3/YY1 in LPS-stimulated THP-1 cells (data not shown). In addition, YY1 acts as an activator that directly binds to the COX-2 promoter and enhances COX-2 expression induced by LPS; however, LPS treatment did not increase the recruitment between YY1 and HDAC1/2 in macrophages [52]. Our finding also described that WK2-16 strongly increased COX-2 expression induced by LPS in THP-1 cells. These findings suggested that WK2-16 suppresses MMP-9 expression and enhances COX-2 expression through influencing both the gene repression and activation of YY1 activity.

In the early response of sepsis, both IL-6 and TNF-α are major pro-inflammatory cytokines and are involved in physiological dysfunction [56,57,58]. Additionally, thermo-dysregulation is mediated by TNF-α because knockout TNF receptor of mice could attenuate hypothermia in early sepsis [56]. Furthermore, COX-2 induction could cause hypothermia, and COX-2 gene deletion could improve hypothermia induced by LPS [59]. WK2-16 has no significant effect on the body temperature of septic mice which might be attributed to that it attenuates the circulating TNF-α level of septic mice but increases monocytic COX-2 expression in vitro*.* Among septic gastrointestinal symptoms, WK2-16 could not improve the body weight loss during sepsis. Additionally, knockout of IL-6 or TNF-α could not affect the loss of body weight during sepsis in mice [56]. Furthermore, blocking IL-6 signaling at an early stage of sepsis provided a therapeutic benefit [60]; consistently septic patients with a lower serum IL-6 concentration showed a low mortality risk [61]. Nevertheless, much evidence has demonstrated that anti-TNF-α therapy could improve septic patient survival [62]. Our findings demonstrated that WK2-16 attenuated both the circulating IL-6 and TNF-α levels in the early stage of sepsis, implying that it could be used as a potential and attenuative therapeutic agent in sepsis. Similarly, both the effect of ITF3056 [27] and WK2-16 indicated HDAC8 inhibition could down-regulate IL-6 expression. It needs to be further investigated whether WK2-16 downregulated IL-6 production via regulating YY1 activity.

## 4. Materials and Methods 

### 4.1. Materials

WK2-16, also called (*E*)-*N*-hydroxy-4-methoxy-2-(biphenyl-4-yl)cinnamide, was obtained from Professor Wei-Jan Huang [45]. Anti-mouse and anti-rabbit immunoglobulin (Ig) G-conjugated horseradish peroxidase (HRP) complexes were purchased from Amersham Biosciences (Sunnyvale, CA, USA) and Jackson-ImmunoResearch (West Grove, PA, USA), respectively. The mouse monoclonal antibody (mAb) specific for human native 92-kDa MMP-9, p38 (total)/phospho-p38 and α-tubulin were purchased from LabVision/NeoMarkers (Fremont, CA, USA). The rabbit pAbs specific for phospho-NF-κB p65 (Ser276 or Ser536), phospho-Akt (Ser473), ERK 1/2 (Total)/phospho-ERK (Thr202/Tyr204), JNK (Total)/pJNK (Thr183/Tyr185), acetyl lysine were purchased from Cell Signaling (Danvers, MA, USA). A rabbit pAb specific for acetyl-histone H3 was purchased from Millipore (Billerica, MA, USA). A rabbit pAb specific for SMC3 and HSP 70 were purchased from Enzo Life Sciences (Plymouth Meeting, PA, USA). A rabbit pAb specific for acetyl-SMC3 was purchased from MBL (Woburn, MA, USA). Normal rabbit Ig G, normal mouse Ig G and rabbit pAb specific for YY1 were purchased from Santa Cruz Biotechnology (Santa Cruz, CA, USA). Thiazolyl blue tetrazolium bromide (MTT), 4-(2-hydroxyethyl)-1-piperazineethanesulphonic acid (HEPES), sodium dodecyl sulfate (SDS), phenylmethylsulfonyl fluoride (PMSF), leupeptin, aprotinin, sodium fluoride, sodium orthovanadate, sodium pyrophosphate, diethyl pyrocarbonate (DEPC), LPS, parthenolide (PTL), phorbol 12-myristate 13-acetate (PMA), and bovine serum albumin (BSA) were purchased from Sigma-Aldrich (St. Louis, MO, USA). Recombinant human TNF-α was purchased from Pepro Tech EC (London, UK). All other chemicals used in this study were of reagent grade.

### 4.2. Cell Cultivation

The acute monocytic leukemia THP-1 cell line was obtained from American Type Culture Collection (Manassas, VA, USA). Cells (passage 12 to passage 30) were cultured in RPMI 1640 medium containing HEPES (18 mM), l-glutamine (3.65 mM), NaHCO3 (23.57 mM), penicillin (90 units/mL), streptomycin (90 μg/mL), and 10% heat-inactivated fetal bovine serum (FBS). Cells were sub-cultured twice per week (1.2 × 10^6^ cells/mL in 75T flask) as previously described [63]. 

### 4.3. Gelatin and Reverse Zymography

THP-1 cells (5 × 10^5^ cells/0.5 mL in 24-well plates) in RPMI 1640/FBS (0.5%) medium were pretreated with or without WK2-16 for 15 min and were subsequently stimulated with LPS (50 ng/mL), TNF-α (10 ng/mL), PMA (2 nM), or TGF-β (10 ng/mL) for 24 h, followed by collection of the supernatants. MMP-mediated gelatinolysis was detected by gelatin zymography. TIMP-1 activity was determined by reverse zymography as previously described [63].

### 4.4. Cellular Viability Assay

THP-1 cells (5 × 10^5^ cells/0.5 mL) were incubated in 24-well plates with different concentrations of WK2-16 at 37 °C for 24 h. The cytotoxic effects of WK2-16 against the THP-1 cells were in relation to vehicle (DMSO)-treated controls that were determined by the MTT assay as previously described [63]. 

### 4.5. Co-Immunoprecipitation and Western Blot Analyses

THP-1 cells (1.0 × 10^6^ cells/mL in six-well plates) in RPMI 1640/FBS (0.5%) medium were pretreated with or without WK2-16 for 15 min and were subsequently stimulated with LPS (50 ng/mL) for the indicated time. Cells were harvested and lysed as previously described [34]. For co-immunoprecipitation (Co-IP), THP-1 cells (14 × 10^6^ cells/14 mL) were incubated with or without WK2-16 or 15 min and were subsequently stimulated with LPS (50 ng/mL) for 2 h. Cells were harvested, washed in ice-cold PBS (pH 7.4), and lysed in lysis buffer (25 mM HEPES, 150 mM NaCl, 5 mM EDTA, 1% Triton X-100, 5 mM sodium pyrophosphate, 1 mM Na_3_VO_4_, 10 mM NaF, 10 μg/mL aprotinin, 10 μg/mL leupeptin, and 1 mM PMSF). Immunoprecipitates were washed three times in lysis buffer and were eluted by boiling in reducing sample buffer. The samples were fractionated by SDS-PAGE and were transferred to PVDF or nitrocellulose membranes. Western blots were probed with the indicated antibodies and then were visualized by enhanced chemiluminescence (PierceTM, Thermo Fisher Scientific, Waltham, MA, USA) and exposure to photographic film (GeneseeScientific, San Diego, CA, USA) or UVP GelDoc-It2 310 Imaging System (Labrepco, Horsham, PA, USA). The films were scanned into Adobe Photoshop, and whole images were adjusted for brightness. The images were cropped and formatted in Adobe Illustrator. The respective fold was analyzed as previously described [63].

### 4.6. Reverse Transcription Polymerase Chain Reaction (RT-PCR) Analysis

THP-1 cells (10^6^/mL in six-well plates) in RPMI164/FBS (0.5%) medium were pretreated with or without WK2-16 for 15 min and were subsequently stimulated with LPS (50 ng/mL). Total RNA was isolated from THP-1 cells stimulated with LPS for 8 h using TRIsure™ (Bioline, Trento, Italy) following the manufacturer’s instructions, and 1 μg of total RNA was reverse transcribed into cDNA using a commercial kit (Super Script On-Step RT-PCR system, GIBCOTM, Thermo Fisher Scientific; Waltham, MA, USA). The nucleotide sequences of the primers used for amplification were as follows: for MMP-9, sense 5′-CGTGG AGAGT CGAAA TCTCT G-3′ and antisense 5′-CCAAA CTGGA TGACG ATGTC T-3′; for GAPDH, sense 5′-CCACC CATGG CAAAT TCCAT GGCA-3′ and antisense 5′-TCTAG ACGGC AGGTG CAAAT CACC-3′. PCR was performed using the following conditions: 28 cycles of a 15-s denaturation step at 94 °C, a 30-s annealing step at 54 °C, and a 60-s extension step at 72 °C for MMP-9; followed by 25 cycles of a 15-s denaturation step at 94 °C, a 30-s annealing step at 67 °C, and a 60-s extension step at 72 °C for GAPDH. The respective amplified PCR products were analyzed as previously described [63].

### 4.7. Transfection and Luciferase Assay

THP-1 cells were co-transfected with the NF-κB reporter plasmid expressing firefly luciferase (pNFB-Luc) (Clontech, Mountain View, CA, USA) and the Renilla luciferase vector pRL-TK (Promega, Madison, WI, USA) using the Effectene transfection reagent (Qiagen, Valencia, CA, USA) as previously described [33]. After 12 h of transfection, THP-1 cells were centrifuged, redispensed in RPMI 1640/FBS (0.5%) medium, and starved for an additional 85 min, and then were pretreated with or without WK2-16 for 15 min and were subsequently stimulated with LPS (50 ng/mL) for 4 h. Cell extracts were prepared using the reporter lysis buffer (Promega), and the luciferase activity was measured by the Dual-Luciferase Reporter Assay System (Promega). The luminescent intensity was determined using an Orion MPL2 microplate luminometer (Berthold Detection Systems, Bleichstrae, Germany) with analytic software (vers. 2.0 R1, Simplicity photon counting, Berthold Detection Systems, Bleichstrae, Germany). All luciferase activities shown in the transient transfection assays were corrected by the activity of Renilla luciferase.

### 4.8. Cytokine Level Measurement by the Enzyme-Linked immunosorbent Assay (ELISA)

THP-1 cells were pretreated with or without WK2-16 for 15 min and were subsequently stimulated with LPS (50 ng/mL) for the described time, followed by collection of the supernatants. The levels of IL-1β and IL-6 released by THP-1 cells were quantified using the ELISA Ready-SET-Go! kit (eBioscience, San Diego, CA, USA) according to the manufacturer’s instructions. The quantitative levels of the cytokines were corrected by the cell number, and the data are shown as picograms (pg)/10^6^ cells.

### 4.9. LPS-Induced Endotoxemia In Vivo

Male ICR mice (8–10 weeks, 25–28 g) were purchased from bioLASCO, Taiwan Co., Ltd. (Taipei, Taiwan). The Institutional Animal Care and Use Committee (IACUC) of Taipei Medical University approved the animal experiments in this study (LAC-101-0130, 1 Aug 2013). Male C57BL/6 mice were assigned to receive LPS (*E. coli* LPS, serotype 0127:B8, 10 mg/kg) or normal saline via intraperitoneal injection. WK2-16 was dissolved in cosolvent as cremophor EL/ethanol (1:1) and was diluted (1:10) with normal saline before injection. Control mice were injected with an equivalent volume of the vehicle (cosolvent). To evaluate the effects of WK2-16 on the pro-inflammatory cytokine production during endotoxemia, WK2-16 (30 mg/kg) was administered 30 min before LPS treatment, and the animals were continuously monitored for 24 h. Whole blood was collected at the indicated times. The serum levels of IL-6 and TNF-α were quantified by ELISA as previously described [63].

### 4.10. Statistical Analyses

The experimental results are expressed as the means ± S.D. accompanied by the number of observations. The results analyzed were performed using one-way analysis of variance (ANOVA) and SigmaStat v3.5 software (SYSTAT Software, San Jose, CA, USA). When group comparisons showed a significant difference, the Student Newman–Keuls test was used. *p* < 0.05 was defined as statistical significance.

## 5. Conclusions

Our finding suggests that WK2-16 suppresses LPS-induced MMP-9 expression by enhancing the recruitment of ac-YY1/u-YY1/HDAC1. In addition, we have found that WK2-16 suppresses IL-6 production from LPS-stimulated THP-1 cells and attenuated the plasma levels of both IL-6 and TNF-α in endotoxemic mice. The detailed mechanisms of the interaction of HDAC8 inhibition and recruitment of YY1 and HDAC1 in MMP-9 and cytokine production in LPS-stimulated THP-1 remain to be further investigated. Taken together, these findings suggested that WK2-16 with HDAC8 inhibition could abrogate MMP-9 and IL-6 production, possibly providing a novel therapeutic strategy of sepsis and systemic inflammation.

## Abbreviations

COX-2	Cyclooxygenase-2
DMSO	Dimethyl sulfoxide
HAT	Histone acetyl-transferases
HDAC	Histone deacetylase
HSP70	Heat shock protein 70
IL	Interleukin
IKK	IκB kinase
LPS	Lipopolysaccharide
MAPK	Mitogen-activated protein kinase
MMPs	Matrix metalloproteinases
PMA	Phorbol 12-myristate-13-acetate
PTL	Parthenolide
SAHA	Suberoylanilide hydroxamic acid
SMC3	Structural maintenance of chromosomes 3
TGF	Transforming growth factor
TIMP-1	Tissue inhibitor of Metalloproteinase-1
TLR4	Toll-like receptor 4
TNF-α	Tumor necrosis factor-α
YY1	Yin Yang 1
